# Epidemiological trends in tuberculosis during a technical assistance project, Lusaka, Zambia, 2018–2021

**DOI:** 10.5588/pha.26.0029

**Published:** 2026-08-24

**Authors:** S. Daka, M. Ota, S. Hirao, G. Samungole

**Affiliations:** 1 Japan Anti-Tuberculosis Association, Lusaka, Zambia;; 2 Research Institute of Tuberculosis, Tokyo, Japan;; 3 National Tuberculosis and Leprosy Programme, Ministry of Health, Lusaka, Zambia.

**Keywords:** tuberculosis, presumptive TB, treatment success rate, TB notifications, treatment supporter

## Abstract

**SETTING:**

Five health facilities in Lusaka, Zambia.

**OBJECTIVE:**

An observational study to describe activities of a technical assistance project in 2019–2021 with baseline epidemiological indicators in 2018.

**RESULTS:**

The number of presumptive tuberculosis (TB) cases at the five sites significantly rose from 7,063 in 2018 to 48,250 in 2021 with a total diagnosis of 9,642 cases of all types of TB and 4,747 cases of bacteriologically positive TB in the 4 years. The percentage of the bacteriologically positive cases among the presumptive TB ones varied from 8.2% to 11.3% in facilities. The proportion of bacteriologically positive TB among the presumptive TB cases at five sites declined from 14.1% in 2018–2019 to 8.3% in 2020–2021 (*P* < 0.001). Treatment success rates maintained high over 85% in four facilities, whereas in the other improved significantly. The ‘not evaluated’ rates of treatment outcomes dropped from 7.0% at all the facilities in 2018–2019 to 1.0% in 2020. During the project, three GeneXpert and a digital X-ray machines were installed in the facilities.

**CONCLUSION:**

Investment in TB, particularly in diagnostic tools, and training of health care workers, coupled with close monitoring of health workers and community volunteers, can greatly improve core TB indicators.

Zambia is among the 30 countries with a high burden of tuberculosis (TB),^1^ and the burden is highest in Lusaka province, the capital city, which accounts for about two fifths of the total TB patients. Exceptional progress has been made, however, in various core TB indicators, which aims at ending TB by 2030 in line with the End TB Strategy.^2^ The sustained increases in TB notifications, treatment success rate, and TB preventive treatment initiations have led to a rapid reduction in the TB incidence rate, which fell from 391 to 272 per 100,000 population between 2015 and 2024.^1^ We have carried out TB and communicable disease control projects in Lusaka^3–11^ and elsewhere.^12–15^ Experiences from these projects are used to improve successive projects aimed at strengthening case finding and management of TB patients.

There is limited evidence on how integrated technical assistance packages influence multiple TB indicators over time. This report describes activities of a technical assistance project at five health facilities in Lusaka, Zambia, with epidemiological indicators from 2019 to 2021, using the data from 2018 as the baseline.

## METHODS

Lusaka district, located within Lusaka province, the capital of Zambia, is the most populated district in the country with a population of 2,212,301 in 2022.^16^ It has 66 public health facilities and we conducted our project between 2019 and 2022 at five health facilities: Chipata First Level Hospital (CFLH, population: 494,670), Chelstone Health Centre (CHC, population: 136,056), Mtendere Health Centre (MHC, population: 88,922), Ng’ombe Health Centre (NHC, population: 75,332), and Kalingalinga Health Centre (KHC, population: 103,721). All five health facilities are TB diagnostic centres, equipped with Gene Xpert machines for TB diagnosis and microscopes for monitoring patients upon treatment, but only CFLH has an X-ray machine.

### Procedures for patient screening, examination and treatment

In Zambia, the Ministry of Health encourages all patients who visit health facilities for various reasons to be screened for TB. The screening is done by asking about the key TB signs and symptoms that include a persistent cough, fever, night sweats, and weight loss.^17^ This activity can be done by community volunteers called TB treatment supporters (TSs) or by health workers.

Apart from patients who admit themselves into the health facilities, sputum samples are also collected from the communities. This is done during community sensitisation programmes where TSs target TB hot spots, provide health education and screening to the public, and collect samples that are taken to the laboratory. The TSs also conduct TB contact investigations of all TB contacts. The Ministry of Health recommends molecular methods such as the use of a GeneXpert system for TB diagnosis.^17^ Such methods are not only highly sensitive but also provide drug resistance status. In the absence of a GeneXpert, microscopes are used. Radiological examination through a chest X-ray can also be a basis for TB diagnosis. Once laboratory results are ready, they are provided to chest clinics. For persons found to have TB, including those who are diagnosed clinically, anti-TB treatment is commenced.

### Project activities

The project undertook the following activities.

#### Training

Various categories of health workers were trained throughout the project life cycle. The aim was to enhance their skills with the expectation that they would offer quality care to the patients. Clinicians were trained in TB management and chest X-ray reading. Radiography technologists were trained in X-ray taking while TB nurses were trained as trainers of TSs. Further, we trained medical equipment technologists in X-ray maintenance and the laboratory personnel in GeneXpert machine general operations and microscopy. The TSs were trained in basic TB management, HIV testing, and counselling.

#### Provision of medical equipment

In the first year of the project, a digital X-ray machine was installed at KHC. The expected outcome was to increase the number of TB notifications by ensuring that all sputum-negative patients went for further evaluation. GeneXpert machines were also installed at CFLH, MHC, and CHC. These would improve detection of bacteriologically positive TB and screening for rifampicin resistance. All these facilities were also equipped with air conditioners.

#### Monitoring and data reviews

We conducted quarterly visits to health facilities in order to monitor TB activities, ensuring that patients were managed according to the national guidelines. Key TB indicators were also monitored, and it was ensured that the registers, the primary facility data collection tools, were filled correctly. Data review meetings were held every quarter to discuss the TB programme performance, track indicators, and propose solutions to the identified gaps. The aim was to improve treatment outcomes.

#### Community activities

Community activities were aimed at creating TB awareness in the communities with the expectation of increasing the number of presumptive TB and notifications. TSs worked both at the facilities and in the community. They carried out TB sensitisation through drama performances and door to door visits, TB screening, and contact investigations, and monitored TB patients.

### Review of data

TB data that were collected during project implementation were reviewed, focusing on numbers and trends for presumptive TB, notifications, treatment success rates, mortality, lost to follow-up, and not evaluated rates for the five health facilities. These variables were chosen and defined in line with the national and WHO TB reporting standards^17,18^: presumptive TB – a patient who presents with symptoms or signs suggestive of TB; treatment success rate – the sum of cured and treatment completed; died – a TB patient who dies for any reason before starting or during the course of treatment; lost to follow-up – a TB patient who did not start treatment or whose treatment was interrupted for two consecutive months or more; not evaluated – a TB patient for whom no treatment outcome is assigned. We examined the treatment outcomes of the patients registered in 2019–2020 with the 2018 data as the baseline. After January 2022 when the project activities were terminated, we were unable to collect data on treatment outcomes for those who were registered in 2021.

#### Data analysis

The data were analysed between pre- and earlier-project periods (2018–2019) and later project period (2020–2021 for case finding and 2020 for treatment outcomes) using Pearson’s *t* test for historical absolute numbers and χ² tests for proportions.

#### Ethical statement

The study was conducted as a part of the routine evaluation activities of a TB project implemented in Lusaka, with the Memorandum of Understanding co-signed by the Ministry of Health of the Republic of Zambia, the Lusaka District Health Office, and the Japan Anti-Tuberculosis Association in February 2018. In compiling the data on case finding and treatment outcomes of patients with TB, the names and addresses of the patients were not collected to protect their confidentiality. The authors obtained a waiver of institutional review for conducting the study from the institutional review board of Research Ethics and Science Converge, Lusaka, Zambia (Ref. No: 2026-Feb-017, dated 18/02/2026). We also obtained permission from the National Health Research Authority of Zambia, Lusaka, Zambia, to publish the study (Ref. No: NHRA-3291/24/02/2026, dated 08/04/2026).

## RESULTS

From 2018 through 2021, a total of 9,642 cases of all types of TB, including 4,747 cases of bacteriologically positive TB, were diagnosed and registered out of 48,250 presumptive TB cases examined in the five facilities (Table). The percentage of the bacteriologically positive cases among the presumptive TB varied from 8.2% in CHC to 11.3% in NHC.

**TABLE.** Number of cases of presumptive, all types of, and bacteriologically positive tuberculosis (TB) by facility, Lusaka, Zambia, 2018–2021.

Health facility	All types of TB		Bacteriologically positive TB		Presumptive TB	
	*n*	%	*n*	%	*n*	%
Chelstone Health Centre	1,809	15.7	944	8.2	11,530	100.0
Chipata Level 1 Hospital	3,364	21.0	1,785	11.1	16,042	100.0
Kalingalinga Health Centre	1,757	22.3	754	9.6	7,844	100.0
Mtendere Health Centre	1,655	20.9	711	9.0	7,924	100.0
Ng'ombe Health Centre	1,057	21.5	553	11.3	4,910	100.0
Total, *n* (%)	9,642	20.0	4,747	9.8	48,250	100.0

**FIGURE 1.**
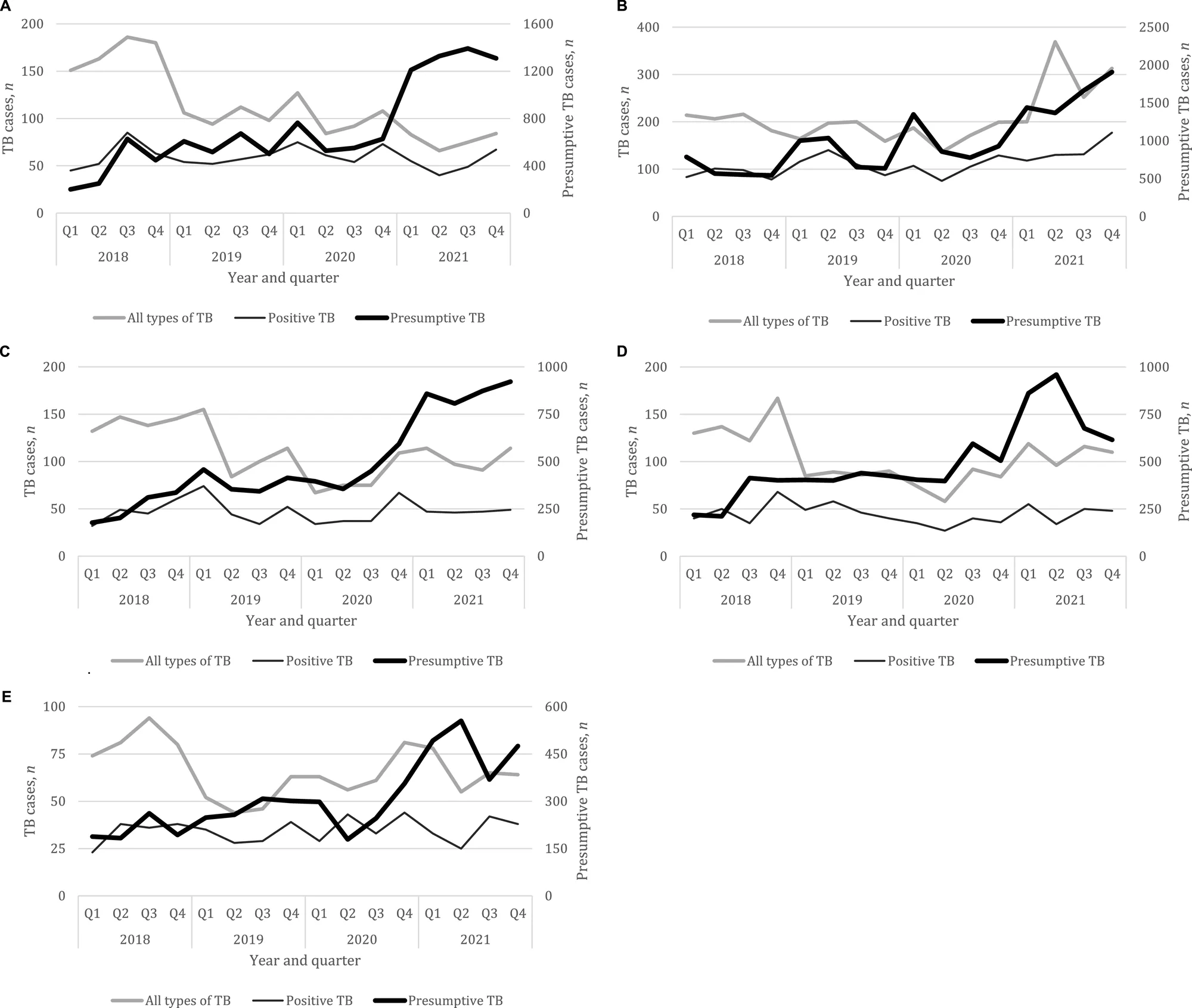
Trends of presumptive tuberculosis (TB), all types of TB, and bacteriologically positive TB in selected health facilities, Lusaka, Zambia, 2018–2021. **A)** Chelstone. Between years 2018–2019 and 2020–2021, the average number of presumptive TB increased from 477.6 per quarter to 963.6 (*P* < 0.01), whereas the number of all types of TB decreased from 136.3 per quarter to 79.7 (*P* = 0.01). The number of bacteriologically positive TB remain unchanged (58.8 vs. 59.3, *P* = 0.93). **B)** Chipata. Between years 2018–2019 and 2020–2021, the average number of presumptive TB increased from 720.4 per quarter to 1,284.9 (*P* < 0.01), whereas the numbers of all types of TB and bacteriologically positive TB remain unchanged (192.1 vs. 228.4, *P* = 0.24 and 101.7 vs. 121.5, *P* = 0.14, respectively). **C)** Kalingalinga. Between years 2018–2019 and 2020–2021, the average number of presumptive TB increased from 324.0 per quarter to 656.5 (*P* < 0.01), whereas the number of all types of TB decreased from 126.9 per quarter to 92.8 (*P* < 0.01). The number of bacteriologically positive TB remain unchanged (48.8 vs. 45.5, *P* = 0.60). **D)** Mtendere. Between years 2018–2019 and 2020–2021, the average number of presumptive TB increased from 363.9 per quarter to 626.6 (*P* < 0.01), whereas the numbers of all types of TB and bacteriologically positive TB remain unchanged (113.3 vs. 93.6, *P* = 0.16 and 48.3 vs. 40.6, *P* = 0.15, respectively). **E)** Ng’ombe. Between years 2018–2019 and 2020–2021, the average number of presumptive TB increased from 242.5 per quarter to 371.3 (*P* = 0.03), whereas the numbers of all types of TB and bacteriologically positive TB remain unchanged (66.8 vs. 65.4 *P* = 0.16 and 33.3 vs. 35.9, *P* = 0.15, respectively).

Figure 1A–E shows the trends of presumptive, all types of, and bacteriologically positive TB in the five facilities from 2018 through 2021. In all the facilities, the numbers of presumptive TB increased between the periods 2018–2019 and 2020–2021. The numbers of all types of TB decreased in CHC and KHC, whereas in others remained almost unchanged. The numbers of bacteriologically positive TB remained unchanged, either, in all the facilities.

The proportion of bacteriologically positive TB among the presumptive TB for all five facilities decreased from 14.1% in the pre-project and the earlier period to 8.3% (*P* < 0.001) in the later period.

**FIGURE 2.**
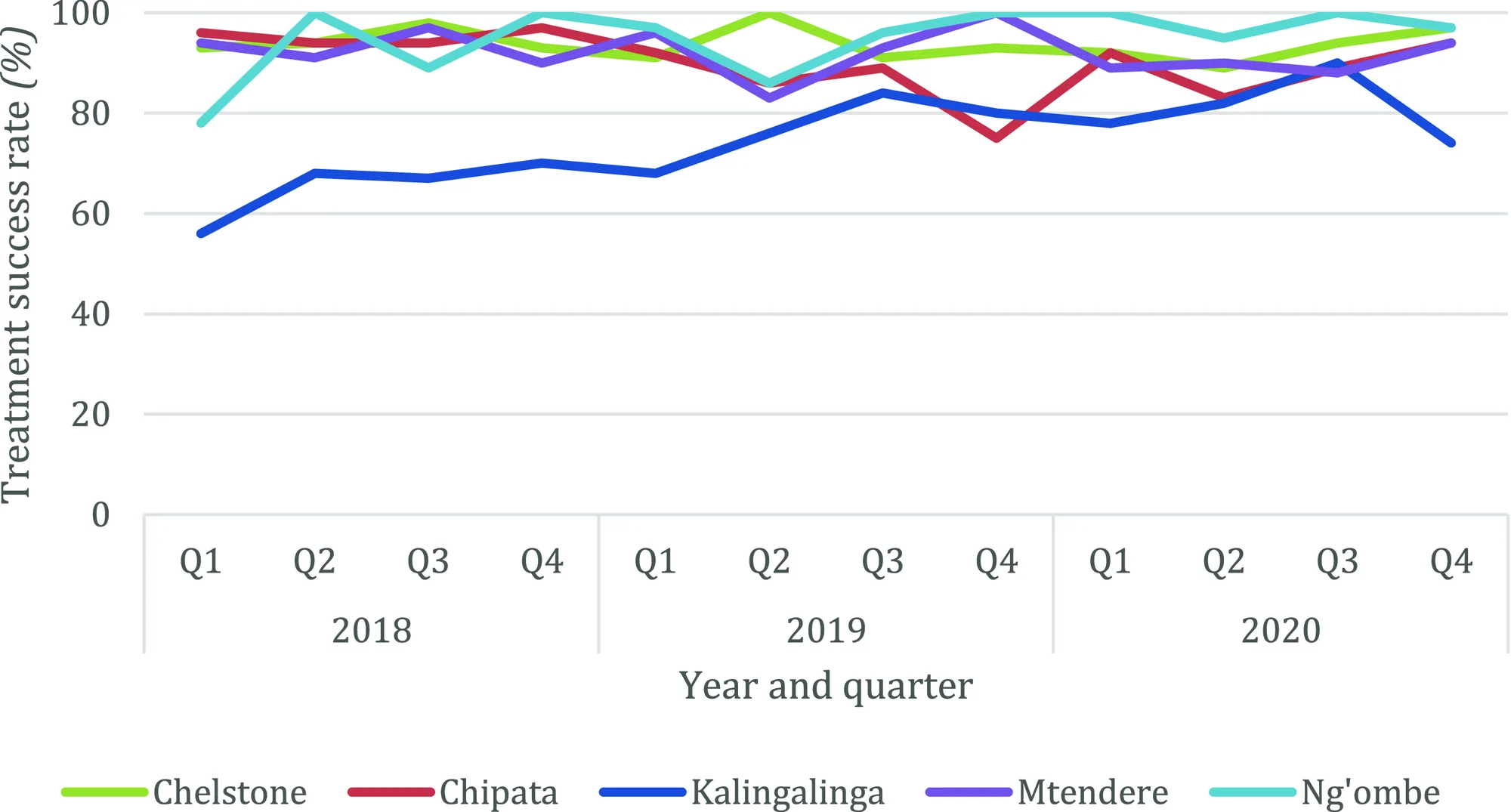
Treatment success rate of bacteriologically positive tuberculosis, Lusaka, Zambia, 2018–2020. The treatment success rates of Kalingalinga improved significantly from 70.4% in 2018 and 2019 combined to 79.7% in 2020 (*P* < 0.05), whereas that of Chelstone, Chipata, Mtendere, and Ng’ombe did not change significantly (*P* = 0.59, *P* = 1.00, *P* = 0.56, and *P* = 0.14, respectively).

Figure 2 presents the trends of treatment success rates for patients with bacteriologically positive TB of which KHC improved significantly from 70.4% in 2018 and 2019 combined to 79.7% in 2020 (*P* < 0.05), whereas that of the other facilities did not change significantly. It should be noted that the treatment success rates of the four facilities except Kalingalinga almost maintained over 85% throughout the project period. In addition, the proportion of ‘not evaluated’ significantly reduced from 7.0% in 2018 and 2019 combined for the five facilities to 0.1% in 2020 (*P* < 0.001), though the fourth quarter of 2019 recognised a sudden pop of ‘not evaluated’ rate to 19.4% in CFLH.

## DISCUSSION

The authors described the project activities at the five health facilities with a review of TB epidemiological indicators of 2019–2021 for comparison with 2018 as a baseline. A total of 48,250 presumptive TB cases were evaluated. Of these, 9,642 cases of all types of TB were registered, including 4,747 cases of bacteriologically positive TB. In the five facilities in general, the numbers of presumptive TB cases increased, resulting in significant decreases in the proportions of bacteriologically confirmed TB among presumptive TB cases from 14.1% in the pre-project and the earlier period to 8.3% in the later period. While the gains may be attributed to many factors, the project played a role through the provision of the Xpert machines to CFLH, MHC, and CHC, and the provision of a digital X-ray machine to KHC. In addition, the provision of training of the health care workers and TSs may have contributed to improved case management in all facilities except CFLH, resulting in a decrease of ‘not evaluated’ rates. Even in CFLH, although there was a sudden increase in the ‘not evaluated’ rate in the fourth quarter of 2019, the rates were essentially under control, being less than 4% in the other facilities.

The increase in the number of presumptive TB cases in all five health facilities may have been due to the heightened project activities. While we acknowledge that other factors could have played a role in this increase, the project put much emphasis on screening of all patients for TB as they visited the health facilities. TSs were placed at various facility entry points and screened patients for TB before they could see the doctors. The increase in presumptive TB could also be attributed to intensified community TB screening by the TSs. Educative drama performances were conducted at marketplaces and these were followed by TB screening. In addition, for every bacteriologically positive TB patient, contact investigation was done by sending TSs to conduct TB screening in the community.

Over the project implementation period, the proportion of bacteriologically positive patients among the presumptive TB patients was reduced significantly. This may have been due to declining numbers of TB cases in the communities as people were being screened, diagnosed, and put on treatment as well as disruptions by COVID-19 among others. However, the increase of the presumptive TB cases in 2021 for almost all facilities may have been because of positive changes in health seeking behaviour. The project also put much emphasis on proper patient management, including patient monitoring, good record keeping, TB data reviews, and supervision of TB nurses. These actions could have played a role in improving treatment success rates for KHC and in almost maintaining high success rates for the other facilities, though CFLH missed twice the target of 85%. It is a high-volume referral hospital with patients coming from beyond Lusaka district, which makes patient monitoring and evaluation difficult.

The findings of this report are similar to those of past studies on strengthening case findings in Lusaka.^5,19^ In Haiti, active TB case finding efforts in high-risk communities and the introduction of new diagnostics contributed to increasing TB case notification.^20^ In Ethiopia, the project-supported zones (i.e., provinces) saw an increase in TB case notification and improved outcomes.^21^ In Uganda, Nigeria, and other areas, interventions were associated with increases in TB notifications.^22–24^

Our study has practical results, showing that with proper health care investment such as provision of medical equipment, training, and close monitoring, it is possible to improve TB indicators. Others can learn from what we did in this project. However, the project was for a specific period of time, and we are not sure if the gains have been sustained after the closure of the project. Additionally, we had no control group and we cannot confirm if all the gains were attributed to the project activities. We also used routinely collected data from facility registers, which are prone to errors.

The results of our project have shown that with investment in medical equipment, training, and close supervision of health workers and TSs, TB indicators can improve. Therefore, we recommend the Ministry of Health and partners to strengthen provision of medical equipment, supervision, and training for health workers. As shown in the reduction in ‘not evaluated’ rates, supervisory visits to health facilities can improve the performance of programmes activities. The Ministry of Health should consider strengthening supervisory visits to health facilities.

## CONCLUSION

There was an increase in the number of presumptive TB cases and improvement in the treatment outcomes. The project contributed to these gains by providing medical equipment, health worker and TS training, supervision, and proper patient management. The National Tuberculosis and Leprosy Programme should prioritise active case finding and training or mentorship of both health workers and TSs and closely monitor health facilities.
